# Saturated Fatty Acid-Enriched Diet-Impaired Mitochondrial Bioenergetics in Liver From Undernourished Rats During Critical Periods of Development

**DOI:** 10.3390/cells8040335

**Published:** 2019-04-10

**Authors:** Aiany C. Simões-Alves, Joao H. Costa-Silva, Idelfonso B. Barros-Junior, Reginaldo C. da Silva Filho, Diogo A. A. Vasconcelos, Hubert Vidal, Béatrice Morio, Mariana P. Fernandes

**Affiliations:** 1 Laboratory of Nutrition, Physical Activity and Phenotypic Plasticity, Federal University of Pernambuco-UFPE, Vitória de Santo Antão, PE 55608-680, Brazil; aiany_simoes@hotmail.com (A.C.S.-A.); joao.hcsilva@ufpe.br (J.H.C.-S.); diogovasconcelos13@yahoo.com.br (D.A.A.V.); 2 Laboratoire de Recherche en Cardiovasculaire, Métabolisme, Diabétologie et Nutrition (CarMeN), INSERM U1060, INRA U1397, Université Claude Bernard Lyon1, 69921 Oullins, France; hubertvidal@yahoo.com (H.V.); beatrice.morio@inra.fr (B.M.); 3 Laboratory of General Biochemistry, Molecular Biology and Exercise, Federal University of Pernambuco-UFPE, Vitória de Santo Antão, PE 55608-680, Brazil; idelfonso1992@hotmail.com (I.B.B.-J.); regis.correia@hotmail.com (R.C.d.S.F.)

**Keywords:** hyperlipidic diet, oxidative metabolism, protein restriction

## Abstract

The nutritional transition that the western population has undergone is increasingly associated with chronic metabolic diseases. In this work, we evaluated a diet rich in saturated fatty acids (hyperlipidic, HL) after weaning of the offspring rats submitted to maternal protein restriction on the hepatic mitochondrial bioenergetics. Wistar rats were mated and during gestation and lactation, mothers received control diets (NP, normal protein content 17%) or low protein (LP, 8% protein). After weaning, rats received either NL (normolipidic) or HL (+59% SFA) diets up to 90 days of life. It was verified that all respiratory states of hepatic mitochondria showed a reduction in the LP group submitted to the post-weaning HL diet. This group also presented greater mitochondrial swelling compared to controls, potentiated after Ca^2+^ addition and prevented in the presence of EGTA (calcium chelator) and cyclosporin A (mitochondrial permeability transition pore inhibitor). There was also an increase in liver protein oxidation and lipid peroxidation and reduction in catalase and glutathione peroxidase activities in the LP group fed HL diet after weaning. Our data suggest that adult rats subjected to maternal protein restriction were more susceptible to hepatic mitochondrial damage caused by a diet rich in saturated fatty acids post-weaning.

## 1. Introduction

According to the World Health Organization, thousands of people, mostly children, die annually or present a delay in physiological development due to nutritional deficiencies and changes in the perinatal environment (during gestation and lactation). Studies have shown that protein reduction during gestation associated with a postnatal environment with increased availability of nutrients seems to induce the early onset of hypertension, type 2 diabetes, dyslipidemia, and arterial hypertension [1,2,3,4]. The underlying mechanisms are based on “phenotypic plasticity” [5,6]. This phenotypic plasticity refers to the ability of an organism to react to changes in the environment with changes in shape, state, movement, or activity rate, without genetic alterations [7].

In the last decades, a new nutritional paradigm has been configured, in which undernutrition has been replaced by overnutrition, characterizing a nutritional transition [8]. This favorable situation for the early onset of chronic-metabolic diseases is strongly associated with the consumption of hypercaloric/hyperlipidic diets [9,10], mainly westernized diets enriched in saturated fatty acids (SFA). In many western countries, the nutritional transition and the inversion of dietary patterns are noteworthy, where there is a high prevalence of excessive food consumption with higher palatability, such as those rich in fats and sugars [11] leading to greater prevalence of overweight and obesity.

The liver during fetal development is very vulnerable to maternal changes, besides being an essential organ in the metabolism of carbohydrates, lipids, and proteins [12]. The literature has reported various effects of protein restriction during critical periods of development on the liver; these include morphological and metabolic alterations, such as content of triglycerides and changes in activity of fatty acid metabolism enzymes, production, and use of energy. Moreover, hepatic dysfunction-related studies have shown that liver injury can be assessed by oxidative stress [13,14,15]. It is believed that oxidative stress is a major cause of developing metabolic disorders such as hepatic steatosis, non-alcoholic fatty liver disease, and type 2 diabetes, associated to mitochondrial dysfunction [16,17,18]. Another study showed that exposure to a diet with 45% lipid content during gestation, lactation, and postnatal periods in the offspring caused changes in the redox state and disturbances in the gene expression related to lipid metabolism in the liver of adult rats [19].

Mitochondria are the main organelles of cellular energy metabolism, responsible for the vast majority of adenosine-5-triphosphate (ATP) synthesis via oxidative phosphorylation. In recent decades, mitochondria have emerged as organelles equipped with sophisticated machinery to mediate the Ca^2+^ flow through the internal mitochondrial membrane and are involved in signaling pathways, injury, and cell death (i.e., apoptosis) [20]. Excess in reactive oxygen species (ROS) production associated with a decrease on its elimination may generate a situation known as oxidative stress [21], often related to mitochondrial dysfunction.

It is well documented that exposure to dietary factors may cause an increase in oxidative stress, making the body vulnerable to reactive oxygen species, mitochondrial dysfunction, reduction of antioxidant genes expression, and related mitochondrial biogenesis [22]. In vitro assays showed that saturated fatty acids may induce oxygen species production related to long-chain fatty-acid metabolism [23,24]. Overall, accumulation of palmitic acid in tissues has been described as a risk factor in developing metabolic and mitochondrial dysfunctions [25]. However, the physiological effects of saturated fatty-acid-enriched diet consumption in malnourished individuals and its repercussion on bioenergetics and mitochondrial function have not been assessed.

Studies show that alterations in mitochondrial biogenesis co-activators such as peroxisome proliferator-activated receptor gamma coactivator 1-alpha (PGC-1α) contribute to the rise of metabolic diseases including diabetes, obesity, cardiomyopathy, Alzheimer’s disease, and Parkinson’s disease [26,27,28]. In addition, some studies have emphasized the role played by the mitochondrial calcium in order to maintain cellular homeostasis in a condition of nutritional transition [29]. In this context, the proteins VDAC, GRP75, MFN2, and CypD have emerged as essential to the control of the calcium flow into the mitochondria and disruption in their transcriptional pattern is involved in calcium accumulation in the mitochondria and cellular damage in metabolic diseases [30,31,32].

Although the literature shows an association between protein restriction and lipid overnutrition, so far, few studies have shown the effects of a nutritional transition from protein restriction to a saturated fatty-acid-enriched diet, focusing on bioenergetics and mitochondrial function. The main aim of this study was to evaluate the effects of a diet rich in saturated fatty acids on the liver mitochondrial bioenergetics from protein-undernourished rats.

## 2. Materials and Methods

### 2.1. Animals

Male and female Wistar rats were provided by Animal Care and Facilities from the Academic Center of Vitória de Santo Antão, UFPE, Brazil. The animals were maintained at a room with controlled temperature (22 ± 1 °C) and light-dark cycle (dark: 18:00–06:00 h). All experimental protocols were approved by the Ethical Committee of the UFPE, Brazil (23076.016634/2017-52). The body weight of animals was recorded at 90 days on an AS-1000 balance (Marte, São Paulo, Brazil), having an error range of 0.01 g. The food consumption was measured at 90 days, calculated from the difference between the offered diet and what remained after 24 h in g/day.

### 2.2. Diets

Three different diets were used in the study, control (normal contents of protein and lipid, NP and NL), low-protein (LP), and SFA-enriched diets (hyperlipidic-HL). Control and low-protein diets were prepared according to the American Institute of Nutrition [33]. The hyperlipidic diet was prepared as described by Ferro Cavalcante, et al. [34] with modifications. Regarding the composition of the macronutrients, the diets presented in g/100g were control (NP and NL) (17% protein, 6% lipid, and 67% carbohydrate; energy density 3.7 Kcal/100g), low-protein (LP) (8% protein, 6% lipid, and 76% carbohydrate; energy density 3.7 Kcal/100g), and hyperlipidic (HL) (22% protein, 15% lipid, and 55% carbohydrate; energy density 4.5 Kcal/g).

### 2.3. Nutritional Protocols

The rats were placed for mating (one male: two female) and confirmation of pregnancy was assessed by the microscopic presence of spermatozoa in the vaginal smear, which was considered the first day of gestation. After, mothers were randomly transferred to individual cages and fed a diet with 17% protein (Control diet, *n* = 5) or with 8% protein (low-protein—LP, *n* = 5) ad libitum during pregnancy and lactation. After birth (24 h), the offspring were grouped into litters with 8 pups. After weaning (postnatal day 22), one or two male offspring from each litter were randomly housed in collective cages (up to four animals per cage). The puppies of each offspring (control and LP groups) were fed with control or with hyperlipidic diet (SFA-enriched) until 90-days-old, where they were euthanized for liver collection.

### 2.4. Collection of Hepatic Tissue, Homogenization, and Protein Dosage

The animals were euthanized by decapitation (at 90 days of life) and hepatic tissue was dissected and stored at −80 °C until use. Hepatic tissue was homogenized in an extraction buffer (50 mM Tris base and 1 mM EDTA, pH 7.4, with addition of 1 mM sodium orthovanadate and 2 mM PMSF). After homogenization, the samples were centrifuged at 1180× *g* at 4 °C for 10 min. The total protein analysis was performed by the Bradford method, with BSA solution (2mg/mL) used as standard [35].

### 2.5. Mitochondria Isolation

Liver mitochondria were prepared by homogenization followed by differential centrifugation [36]. After decapitation, the rat’s liver was removed immediately and homogenized in a mixture containing 125 mM sucrose, 10 mM HEPES (pH 7.2), 65 mM potassium chloride, 2 mM potassium phosphate, and 1 mM magnesium chloride. The homogenate was centrifuged at 461× *g* for 10 min at 4 °C, and the resulting supernatant was carefully removed and centrifuged at 4722× *g* for 10 min at 4 °C. The supernatant was discarded, and the pellet resuspended in 250 mM sucrose, 5 mM HEPES (pH 7.2), and 0.3 mM EGTA and centrifuged as in previous condition. The pellet containing isolated mitochondria was resuspended in a buffer containing 250 mM sucrose and 5 mM HEPES (pH 7.2). Mitochondrial protein concentration was determined spectrophotometrically (Biochrom LIBRA S12 UV/VISIBLE, Biochrom Ltd., Holliston, MA, USA) according to Bradford [35].

### 2.6. Mitochondrial Oxygen Consumption

Measurement of mitochondrial respiration was performed at 28 °C in a closed thermostatic glass chamber connected to a Clark-type oxygen electrode (Hansatech Instruments, Pentney King’s Lynn, UK) as described previously by Robinson and Cooper [37]. Mitochondria were suspended at a concentration of 0.5 mg of protein/mL in respiration buffer containing 125 mM sucrose, 10 mM HEPES (pH 7.2), 65 mM KCl, 2 mM K_2_HPO_4_, 1 mM MgCl_2_, 2 µM rotenone, 5 mM succinate, and 0.5 mM EGTA. Mitochondrial respiration was measured with Complex II substrate. The following were added to the cell respiration experiments: ADP (200 µM), oligomycin (1 µg/mL), and CCCP (1 µM).

### 2.7. Mitochondrial Permeability Transition Pore (MPTP) Opening

MPTP was determined as described previously [38]. Opening of the pore induces mitochondrial swelling, which is measured spectrophotometrically (Biochrom LIBRA S12 UV/VISIBLE, Biochrom Ltd.) as a reduction in absorbance at 520 nm. Isolated mitochondria (0.5 mg/mL of protein) were added into swelling buffer that contained 125 mM sucrose, 10 mM HEPES (pH 7.2), 65 mM KCl, 2 mM K_2_HPO_4_, 1 mM MgCl_2_, and 5 mM succinate. The confirmation of mitochondrial volume increase as a consequence of MPTP was performed through the use of 0.1 µM cyclosporin A (CsA), classical inhibitor of the mitochondrial permeability transition pore, and 0.5 mM EGTA, a calcium chelator.

### 2.8. Oxidative Stress Evaluation in Liver

#### 2.8.1. Evaluation of Substances Reactive to Thiobarbituric Acid (TBARS) Levels

For the dosage of TBARS, the colorimetric technique of Buege and Aust [39] was used. An aliquot (0.3 mg/mL) of the liver homogenate was added to 30% trichloroacetic acid (TCA) and Tris-HCl (10 mmol/L) followed by thorough mixing and centrifugation at 1180× *g* for 10 min. The supernatant was transferred to another tube and 0.73% TBA (*v*/*v*) was added before mixing and boiling for 15 min. The pink pigment yield was then measured at 535 nm in a spectrophotometer (Biochrom LIBRA S12 UV/VISIBLE, Biochrom Ltd.) at RT and expressed as mmol/mg of protein.

#### 2.8.2. Evaluation of Protein Oxidation

The protein oxidation was assessed using the procedures highlighted by Zanatta, et al. [40]. With the samples on ice, 30% (*w*/*v*) TCA was added to the sample (0.3 mg/mL) and then centrifuged for 15 min at 1180× *g*. The pellet was re-suspended in 10 mM 2,4-dinitrophenylhydrazine (DNPH) and immediately incubated in a darkroom for 1 h with agitation every 15 min. Samples were washed and centrifuged three times in ethyl/acetate buffer and the final pellet was re-suspended in 6 M guanidine hydrochloride, incubated for 5 min at 37 °C, and the absorbance read in a spectrophotometer (Biochrom LIBRA S12 UV/VISIBLE, Biochrom Ltd.) at 370 nm. The results were expressed as mmol/mg protein.

#### 2.8.3. Superoxide Dismutase (SOD) Assay

SOD determination was performed in accordance with the protocol developed by Misra and Fridovich [41]. In brief, 0.1 mg of protein was added to a 0.05 M Carbonate buffer with 0.1 mM EDTA, pH 10.2. The reaction was started with 150 mM epinephrine and the SOD activity at 37 °C was measured by the kinetics of inhibition of 1 epinephrine auto-oxidation at 480 nm. One unit of SOD was defined as the amount of protein required to inhibit the auto-oxidation of 1 µmol de epinephrine per minute. The results were expressed in U/mg protein.

#### 2.8.4. Catalase (CAT) Assay

CAT activity was monitored according to Aebi [42]. Briefly, 0.3 M hydrogen peroxide (H_2_O_2_) was added to the sample (0.08 mg of protein) followed by addition of the 50 mM phosphate buffer, pH 7.0 at 20 °C. The decay curve absorption was monitored for 100 s at 240 nm. One unit of CAT was defined as the amount of protein required to convert 1 µmol of H_2_O_2_ per minute to H_2_O. The results were expressed in U/mg protein.

#### 2.8.5. Glutathione Peroxidase (GPX) Assay

GPx activity was performed in accordance with Paglia and Valentine [43]. Briefly, 0.1 mg of protein was added to a 50 mM phosphate buffer, pH 7.0, containing 5 mM EDTA; 0.28 mM NADPH; 3.75 mM sodium azide; 5 mM glutathione reduced (GSH); and glutathione reductase, acquired from Sigma (St. Louis, MO). The reaction was started with 2.2 mM H_2_O_2_. NADPH oxidation followed at 340 nm absorbance at 20 °C and its coefficient of extinction was used to determine the GPx activity as U/mg protein. One unit of GPx was defined as the amount of protein required to oxidize1 µmol of NADPH per minute, based on its molecular absorbance.

#### 2.8.6. Glutathione S-Transferase (GST) Assay

The liver homogenate was used to measure GST activity according to the method of Habig et al. [44] by determination of absorbance at 340 nm after addition of 1 mmol/L of 1-chloro-2.4-dinitrobenzene (CDNB). GST activity was calculated using a 2.4-dinitrophenyl-S-glutathione (DNP-SG) substrate at 30 °C. GST activity was expressed as U/mg of protein. Based on its molecular absorbance, 1 enzymatic unit was defined as the amount of protein required to produce 1 μmol/L DNP-SG per minute.

#### 2.8.7. Glutathione Reduced (GSH) Levels

To determine GSH levels, the liver homogenate (0.1 mg protein) and the o-Phthaldialdehyde (OPT) fluorescent (1 mg/mL) were added in 0.1M phosphate buffer containing 5 mM EDTA (pH 8.0) and incubated at room temperature for 15 min. Fluorescence intensity was measured at 350 nm excitation and 420 nm emission wavelengths in Spectrofluorimeter (Varioskan Flash, Thermo Scientific, Vantaa, Finland) and compared with a known standard GSH curve according to the method of Hissin and Hilf [45]. The results were expressed as μmol/mg of protein.

#### 2.8.8. Evaluation of Total Thiols (SH) Groups

The sulfhydryl content was determined by reaction with DTNB (5,5′-dithiobis (2-nitrobenzoic acid) as described by Ellman [46]. Liver homogenate was incubated in the dark after addition of 10 mM DTNB and the final volume was completed to 1 mL with an extraction buffer (pH 7.4). The absorbance reading was then made in a spectrophotometer at 412 nm (Biochrom LIBRA S12 UV/VISIBLE, Biochrom Ltd.). The results were expressed as μmol/mg of protein.

#### 2.8.9. RNA Extraction, Reverse Transcription, and Quantitative PCR (qPCR)

Total RNA was extracted with Tripure reagent (Roche, Meylan, France) according to the manufacturer′s instructions. Briefly, 10 µL of Trizol were used per mg of liver tissue and the resulting suspension was homogenized using a Precellys Lysing kit (Bertin, Montigny-le-Bretonneux, France). After grinding, ¼ volume of chloroform was added, the preparation vortexed 3 × 15 s, incubated at room temperature for 5 min, and centrifuged for 15 min at 15,000× *g* at 4 °C. The RNA was precipitated by addition of 1/2 volume of isopropanol (Carlo Erba reagents, Val-de-Reuil, France) and centrifugation (15 min at 15,000× *g* at 4 °C). RNA-containing pellets were washed sequentially with 70% and 95% ethanol (Carlo Erba reagents), dried, and dissolved in 100 µl of RNase-free water. RNA concentration and purity (260/280 nm absorbance ratio) was determined on a NanoDrop™ 2000/2000c Spectrophotometer (Thermo Scientific, Wilmington, DE, USA).

Reverse transcription was performed using a RT-TAKARA kit (Primescript TM, Dalian, China) to generate cDNA for Real Time PCR. The quantitative amplification (qPCR) was measured by Rotor-Gene Real-Time PCR System (Labgene Scientific Instruments, Archamps, France). All results are represented as arbitrary units (A.U.) derived from a standard calibration curve derived from a reference sample. A PCR for each sample was carried out in duplicate for all genes and TBP was used as a housekeeping gene. As a further control, qPCR amplicons were analyzed by electrophoresis on 1% agarose gel (data not shown). Sequences of primers used in this study are reported in Table 1.

### 2.9. Statistical Analysis

For statistical analysis, GraphPad Prism^®^ 6.0 for Windows was used, and the results were expressed as mean ± SEM (standard error of the mean). Kolmogorov–Smirnov and Shapiro–Wilk normality tests were applied to all groups. Two-way ANOVA™ test was used to compare all the groups followed by Bonferroni post-test. The level of significance was considered *p* < 0.05.

## 3. Results

### 3.1. Body Weight and Food Consumption

The body weight at 90 days was reduced in LP-NL and LP-HL when compared to NP-NL (NP-NL 371.2 ± 7.2 vs. LP-NL 317.4 ± 5.1 g (−14.5%, *p* < 0.0001), and vs. LP-HL 328.9 ± 11.9 g (−11.4%, *p* < 0.01)). In daily food consumption, a decrease was observed in LP-NL when compared to NP-NL (LP-NL 15.5 ± 0.5 vs. NP-NL 20.0 ± 1.4 g (−22.5%, *p* < 0.001)); while, NP-HL showed an increased food consumption when compared to LP-HL (NP-HL 24.3 ± 0.2 vs. LP-HL 18.4 ± 0.5 g (+24.3%, *p* < 0.001)).

### 3.2. Mitochondrial Respiration

**Figure 1:**
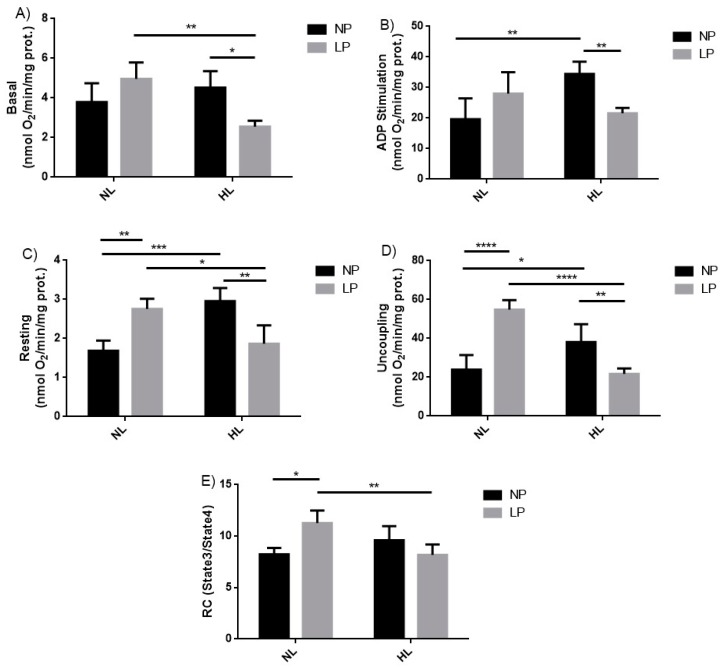
Evaluation of mitochondrial respiration in adult rats subjected to low protein diet during gestation and lactation and post-weaning hyperlipidic diet. Mitochondrial respiration on the basal state (**A**), with ADP (**B**) stimulus, resting state (**C**), uncoupling state (**D**), and respiratory control (**E**), in liver homogenate of male 90-day-old rats born to mothers fed on normoprotein (NP) or low protein (LP) diets, during gestation and lactation, and normolipidic (NL) or hyperlipidic diets (HL) after weaning up to adulthood. *n* = 6–9 animals per group. All values were expressed as mean ± SEM. *p < 0.05; **p < 0.01, ***p < 0.001, ****p < 0.0001 performed with two-way ANOVA followed by the Bonferroni multiple comparison test.

The mitochondrial respiration during basal state was reduced in LP-HL when compared to LP-NL (−43.7%, *p* < 0.05) and NP-HL groups (−48.7%, *p* < 0.01) (Figure 1A); with ADP stimulated (state 3), HL diet was able to increase ADP phosphorylation (NP-NL vs. NP-HL, +43.01%, *p* < 0.01). However, the association of diets during different times led to a decrease in this parameter (LP-HL vs. NP-HL, −37.3%, *p* < 0.01) (Figure 1B). In the resting condition (state 4), there was an increase in LP-NL (+63.1%, *p* < 0.01) and NP-HL (+75.1%, *p* < 0.001) compared to respective control group (NP-NL); and a decrease in LP-HL when compared with LP-NL (−32.1%, *p* < 0.05) and NP-HL groups (−36.8%, *p* < 0.01) (Figure 1C). Similarly, on the uncoupled state, the LP-NL (+128.9%, *p* < 0.001) and NP-HL (+59.1%, *p* < 0.05) groups were higher than NP-NL; and there was a decrease in LP-HL when compared with the NP-HL (−43.0%, *p* < 0.01) and LP-NL (−60.4%, *p* < 0.001) groups (Figure 1D). Respiratory control was significantly higher in the LP-NL compared to NP-NL (+36.8%, *p* < 0.05) and lower between the LP-HL group compared with the LP-NL group (−25.7%, *p* < 0.01) (Figure 1E).

### 3.3. Mitochondrial Swelling

**Figure 2:**
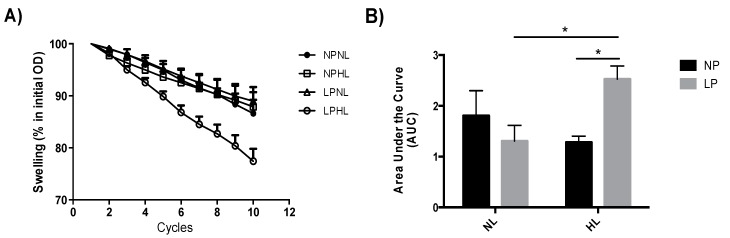
Evaluation of mitochondrial swelling in adult rats subjected to the low protein diet during gestation and lactation and post-weaning hyperlipidic diet. (**A**) Mitochondrial swelling (% of initial OD) and (**B**) area under the curve in mitochondria isolated from liver of 90-day-old male rats born to mothers fed a normoprotein (NP) or low protein (LP), during gestation and lactation, and normolipidic (NL) or hyperlipidic diets (HL) after weaning up to adulthood. *n* = 6–9 animals per group. All values were expressed as mean ± SEM. *p < 0.05 performed with two-way ANOVA followed by the Bonferroni multiple comparison test.

The mitochondrial swelling data revealed that in the presence of calcium ion (10 μM), the area under curve of the LP-HL was higher than the NP-HL (+49.2%, *p* < 0.05) and LP-NL groups (+67.1%, *p* < 0.01) (Figure 2A). These findings indicate a higher swelling of the organelle in animals submitted to a low protein diet during gestation and lactation and a post-weaning HL diet (Figure 2B). After addition of higher calcium concentrations (20 μM), the effect of swelling in the LP-HL group was potentiated (272.1%, *p* < 0.01). It was observed that, in the presence of CsA and EGTA, the mitochondrial swelling was prevented (LP-HL = 2.525 ± 0.25 vs. LP-HL + CsA = 1.112 ± 0.26 (−56%, *p* < 0.0001) and LP-HL = 2.525 ± 0.25 vs. LP-HL + EGTA = 1.212 ± 0.12 (−52%, *p* < 0.001)).

### 3.4. Oxidative Stress Biomarkers

**Figure 3:**
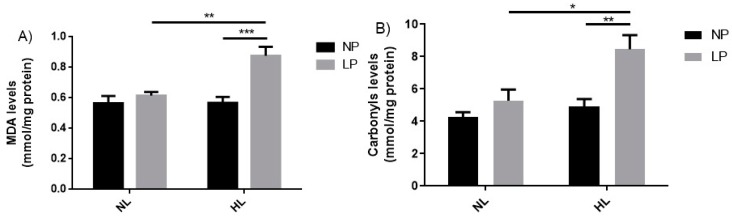
Evaluation of levels of oxidative stress biomarkers in adult rats subjected to hypoproteic diets during gestation and lactation and post-weaning hyperlipidic diets. (**A**) Levels of malondialdehyde and (**B**) levels of carbonyls in liver homogenate of 90-day-old male rats born to mothers fed a normoprotein (NP) or low protein (LP), during gestation and lactation, and normolipidic (NL) or hyperlipidic diets (HL) after weaning up to adulthood. *n* = 5–7 animals per group. All values were expressed as mean ± SEM. *p < 0.05; **p < 0.01; ***p < 0.001; performed with two-way ANOVA followed by the Bonferroni multiple comparison test.

The biomarkers of oxidative stress showed that low protein diet during gestation and lactation followed by a hyperlipidic diet after weaning increased the levels of malondialdehyde (MDA) in LP-NL vs. LP-HL groups (+42.5%, *p* < 0.01) as well as in NP-HL vs. LP-HL groups (+54.3%, *p* < 0.001) (Figure 3A). Carbonyl levels increased in LP-NL vs. LP-HL (+72.2%, *p* < 0.01), as also in NP-HL vs. LP-HL groups (+60.07%, *p* < 0.05) (Figure 3B).

### 3.5. Enzymatic and Non-Enzymatic Antioxidant Responses

**Figure 4:**
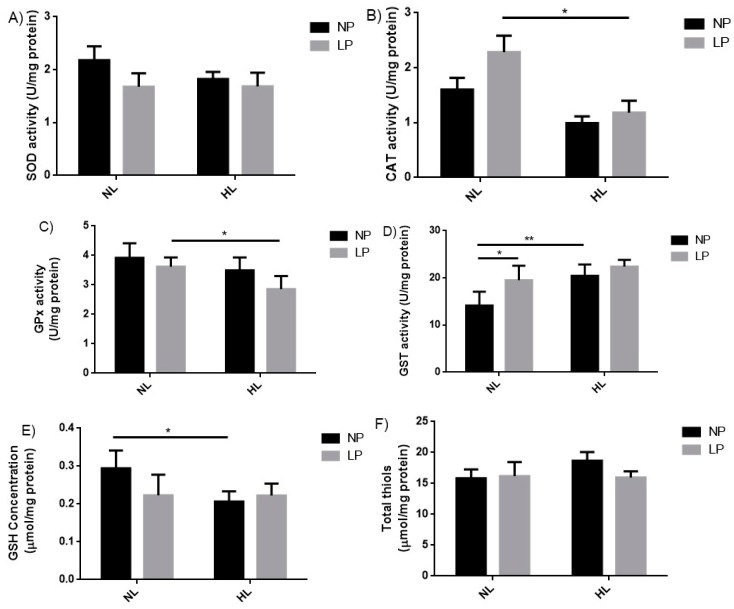
Evaluation of enzymatic and non-enzymatic antioxidant systems in adult rats subjected to the low protein diet during gestation and lactation and post-weaning hyperlipidic diet. (**A**) Superoxide dismutase activity (SOD), (**B**) catalase activity (CAT), (**C**) glutathione peroxidase activity (GPx), (**D**) glutathione-S-transferase (GST) activity, (**E**) reduced glutathione levels (GSH), and (**F**) total thiols in liver homogenate of 90-day-old male rats born to mothers fed a normoprotein (NP) or low protein (LP), during gestation and lactation, and normolipidic (NL) or hyperlipidic diets (HL) after weaning up to adulthood. *n* = 5–7 animals per group. All values were expressed as mean ± SEM. *p < 0.05; **p < 0.01, performed with two-way ANOVA followed by the Bonferroni multiple comparison test.

The evaluation of the enzymatic antioxidant response revealed that the nutritional manipulations did not significantly change the SOD activity (Figure 4A). However, it was observed that CAT (−48.1%, *p* < 0.05) and GPx (−21.0%, *p* < 0.05) were found reduced in LP-NL vs. LP-HL groups (Figure 4B,C, respectively). The GST activity showed an increase when comparing NP-NL with LP-NL (+38.1%, *p* < 0.05) and NP-NL with NP-HL (+44.5%, *p* < 0.01) groups (Figure 4D). HL diet led to a decrease in GSH levels in NP-HL (−29.7%, *p* < 0.05) related to control group (NP-NL) (Figure 4E). There was no significant change in the total thiol levels (Figure 4F).

### 3.6. Gene Expression by RT-PCR

**Figure 5:**
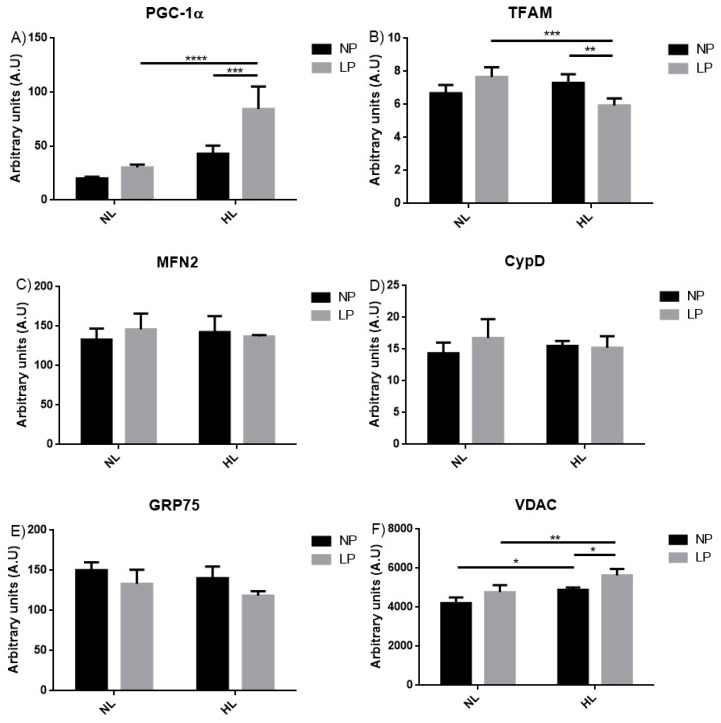
Evaluation of liver gene expression of adult rats subjected to the low protein diets during gestation and lactation and post-weaning hyperlipidic diet. Genic expression of (**A**) Peroxisome proliferator-activated receptor gamma, coactivator 1 alpha (PGC-1α), (**B**) transcription factor A, mitochondrial (Tfam), (**C**) Mitofusin 2 (MFN2), (**D**) Cyclophilin D (CypD), (**E**) glucose-regulated protein 75 (GRP75), and (**F**) voltage-dependent anion channel 1 (VDAC) in liver of 90-day-old male rats born to mothers fed a normoprotein (NP) or low protein (LP), during gestation and lactation, and normolipidic (NL) or hyperlipidic diets (HL) after weaning up to adulthood. All values were expressed as mean ± SEM. *p < 0.05; **p < 0,01; ***p<0.001; ****p < 0.0001, performed with two-way ANOVA followed by the Bonferroni multiple comparison test.

PGC-1α expression was higher in LP-HL when compared to LP-NL (+178.1%, *p* < 0.0001) and LPHL (+95.9%, *p* < 0.001) groups (Figure 5A). Conversely, Tfam showed a decrease between LP-HL vs. LP-NL (−22.5%, *p* < 0.001) as also between the LP-HL vs. LP-NL (−18.6%, *p* < 0.01) groups (Figure 5B). Gene expression of MFN2, CypD and GRP75 did not show differences among groups (Figure 5C–E, respectively). In the VDAC analysis, there were increases comparing the NP-HL vs. NP-NL groups (+16%, *p* < 0.05), LP-NL vs. LP-HL (+18.0%, *p* < 0.01), as well between LP-HL vs. NP-HL (+15.1%, *p* < 0.05) (Figure 5F).

## 4. Discussion

In the last decades, a new nutritional paradigm has been identified, in which undernutrition has been replaced by overnutrition, characterizing a nutritional transition. This has been associated to the consumption of westernized diets enriched in saturated fatty acids (SFA), leading to increased risk factor for chronic-metabolic diseases in adulthood [8]. In the present study, we evaluated the impact of a saturated fatty-acid-enriched diet in the post-weaning offspring subjected to maternal protein restriction on the liver mitochondrial bioenergetics.

In our study, it was observed that offspring from groups feeding on normal protein diets showed high food intake consumption, with direct association with body weight gain. The body weight from low protein groups that received or not a diet rich in saturated fatty acids were reduced in relation to the control group (NPNL). These data confirm the impact protein malnutrition in early life on the growth [47,48] and reveal that a post-weaning consumption of saturated fatty-acids-enriched diet was not able to induce overweight in juvenile rats. It is well known that hyperlipidic diets may impact the growth and fat deposition in adulthood. However, at 90 days of life, no sign of overweight was noted. Thus, all results related to hepatic mitochondrial bioenergetics shown in the present study were observed before the progressive dysfunction in body weight.

The significant increase on the respiratory control ratio (RCR) of rats’ liver mitochondria submitted to a low protein diet during gestation and lactation showed higher electron transport chain activity, as a form of metabolic adaptation to the nutritional insult suffered at early life of these animals. This result corroborates a previous study conducted on the offspring from rats subjected to protein restriction during gestation that showed increase in oxygen uptake on isolated rat liver mitochondria on 45, 60, and 120 days of life [49]. However, when the animals were fed with a diet rich in SFA, we observed a slower mitochondrial respiration rate and RCR suggesting mitochondrial dysfunction (Figure 1).

The literature has shown that inner mitochondrial permeability induced by the calcium ion can be associated with a nonspecific increase in membrane permeability that increases respiratory rates and decreases the coupling between oxygen consumption and oxidative phosphorylation [20,50]. Mitochondrial swelling may be due to mitochondrial cristae unfolding, as result of physiological response, or occurs by opening the mitochondrial permeability transition pore (MPTP), a high-conductance channel that can induce loss of mitochondrial membrane potential, impairment of cellular calcium homeostasis, oxidative stress, and a decrease in ATP production upon pathological activation [51]. The mitochondrial swelling experiments showed that animals that received a low protein diet during gestation and lactation were more resistant to the swelling induced by calcium ion; but when the animals were submitted to low protein diet and a SFA-enriched diet post-weaning up to adulthood, a higher swelling of the organelle (Figure 2) was observed. This effect was potentiated after the addition of higher calcium concentrations. Generally, mitochondrial impairment is triggered by calcium deregulation that can lead to MPTP opening and cell death [51,52]. After the addition of cyclosporin A (mitochondrial permeability transition pore inhibitor) and EGTA (calcium chelator), the mitochondrial swelling was totally prevented, showing that the increased mitochondrial volume observed in the LP-HL was due to the MPTP opening. The MPTP has been considered a key contributor to cell death, inducing neurodegenerative diseases such as Alzheimer’s, Parkinson, and Huntington’s diseases [53], beside cardiomyopathies [54], some types of cancer [55], diabetes [56], and nonalcoholic steatohepatitis [57].

Adequate perinatal nutrition is important because macro- and/or micronutrient deficiency during critical periods of development increases oxidative stress by the generation of reactive oxygen species (ROS) and decreases antioxidant activity associated with offspring insulin resistance [58,59]. Our results showed that protein restriction during gestation and lactation did not alter the oxidative stress biomarkers levels in liver of adult rats. However, when these animals were submitted to a diet rich in SFA from the post-weaning period to adulthood, there was an increase in the lipid peroxidation and protein oxidation levels (Figure 3). A high-fat diet (HFD) is shown to increase free radical production that may generally lead to systemic production of oxidative stress [60] and generate hepatic steatosis in rodents and humans by increased hepatic lipid uptake [61,62].

Under normal developmental conditions, ROS production is balanced by the removal of free radicals by antioxidant mechanisms [63]. Oxidative stress occurs when ROS generation exceeds the scavenging capacity of cellular antioxidant mechanisms as a result of excessive ROS production and/or inadequate antioxidant intake or synthesis [58]. The literature relates that inadequate maternal nutrition and other challenges during development increase maternal and fetal oxidative stress and are responsible for onset and progression of metabolic diseases [64,65,66].

The evaluation of antioxidant systems showed a decrease in CAT and GPx activities in the animals that received HL post-weaning (Figure 4C). Lower CAT activity increases hydrogen peroxide levels and can cause cellular damage [67]. Our results corroborate Tanrikulu Küçük, et al. [68] who also observed a decrease in CAT activity and GPx expression in rat’s liver fed with HFD during 16 weeks. Another study demonstrated that catalase deficiency did not cause noticeable changes in mice phenotype up to 10 weeks of age, but accelerated HFD-induced systemic and liver insulin resistance, liver inflammation, along with increased oxidative stress as early as after two weeks of HFD feeding [69]. Data from the literature also show that GPx depletion induces lipid peroxidation in various tissues, including the liver [70], and is associated to hepatocellular carcinoma [71]. The glutathione-S-transferase (GST), which is an important antioxidant enzyme, acts on the xenobiotics metabolism, participates in redox signaling because it regulates a lipid peroxidation product, 4-hydroxy-trans 2-nonenal (HNE) further involved in the modulation of gene expression, cell proliferation, and apoptosis [72]. Our data showed that independent of nutritional insult, protein restriction, or HL diet, the GST activities were higher than in the control groups (Figure 4D). These results may be related to increase liver activity as a form of metabolic compensation.

The non-enzymatic antioxidant system was evaluated by GSH dosage, a non-protein thiol with a wide range of antioxidant properties that can eliminate O_2_^-^ and OH^-^ radical non-enzymatically, regenerate other antioxidants to their active form, and can be conjugated and excreted with toxins through the reaction catalyzed by glutathione S-Transferases (GST) [64]. GSH levels were decreased in animals that received HL diet post weaning (Figure 4), which suggests a reduction in the non-enzymatic antioxidant system. Intracellular GSH homeostasis is affected by increased ROS production, being associated with alcoholic and non-alcoholic liver diseases [73]. Increased lipid and protein oxidations observed in present study may be considered good biomarkers of cellular homeostasis and suggest that the oxidative balance might be impaired in animals subjected to perinatal low protein and post-weaning HL diets. These data reinforce the hypothesis that subjects exposed to nutritional transition are unable to restore oxidative homeostasis. At least in part, changes in oxidative gene transcription and expression should be involved in these functional effects. However, this hypothesis was not tested in the present study and may be evaluated in future studies.

Nutritional deficiency may affect several essential metabolic processes and can interfere with genome stability. There is evidence suggesting that a reduction in the basal metabolic rate, which could be caused by the decreased rate of cellular oxygen consumption, mitigate DNA damage arising from oxidative stress [74]. Members of the peroxisome proliferator-activated receptor gamma coactivator 1 (PGC-1) family of coactivators have been revealed as key players in the regulation of the energy metabolism. PGC-1 coactivators coordinate the activity of transcription factors to modulate energy metabolism and other cellular processes in response to a variety of environmental and physiological signals [75]. Mitochondrial biogenesis is regulated to adapt the mitochondrial population to cell energy demands. The mitochondrial transcription factor A (Tfam) performs several functions for mtDNA and interactions between Tfam and mtDNA participate to regulation of mitochondrial biogenesis [76]. Our results showed higher PGC-1α expression and Tfam decrease in the animal’s liver submitted to protein restriction during critical periods of development and then received a SFA-enriched diet from the post weaning to adulthood (Figure 5A,B). Initially described as key regulators of the process of mitochondrial biogenesis, recent studies using genetically engineered animal models have uncovered new functions for PGC-1 coactivators beyond the regulation of gene networks strictly related to mitochondrial oxidative metabolism. Lipid synthesis, lipoprotein secretion, muscle fiber type specification, angiogenesis, brite/breige adipocyte differentiation, hematopoiesis, and the immune response are among the new cellular and physiological processes that have been described to be regulated by members of the PGC-1 family [75]. Our Tfam results corroborate the study by Sheldon, et al. [77] that showed lower Tfam expression in the liver of rats fed with HFD (45% fat, 35% carbohydrate and 20% protein) post-weaning. However, in the present study, a mild hyperlipidic diet with 33% fat and enriched in saturated fatty acids was able to induce modification of genes related to mitochondrial biogenesis, such as Tfam and PGC-1. These results support the notion that saturated fatty acids have an impact on mitochondria, especially those subjected to protein restriction during fetal development.

Mitochondrial calcium handling is essential to maintain cellular homeostasis under the condition of nutritional transition. In this context, proteins VDAC, GRP75, MFN2, and CypD have emerged as essential to the control of the calcium flow into the mitochondria. Disruption in their transcriptional pattern have been involved in calcium accumulation in the mitochondria and cellular damage in metabolic diseases.

The voltage-dependent anion channel 1 (VDAC1), located in the outer mitochondrial membrane (OMM), serves as a mitochondrial gatekeeper, controlling the metabolic and energy cross talk between mitochondria and the rest of the cell. VDAC1 is highly Ca^2+-^ permeable, transporting Ca^2+^ to the inter-membranes space and thus modulating Ca^2+^ access to Ca^2+^ transporters in the inner mitochondrial membrane [31]. VDAC plays key role in the interactions between mitochondria with endoplasmic reticulum (ER). This interaction is very important to mitochondrial calcium handling in order to maintain cellular homeostasis, especially under conditions of nutritional transition. Other proteins are implicated in this process, such as GRP75, MFN2, and CypD. The GRP75 is part of the complex involved in ER and mitochondria Ca^2+^ flow and is composed of the inositol-1,4,5-triphoaphate receptor (IP3R) in the ER membrane and of the VDAC at OMM, and coupled by the GRP75 [78]. It is known that reduction in GRP75 expression affects interaction between organelles and impairs mitochondrial calcium handling. Our results showed increased VDAC transcription in the liver of animals that received an HL diet enriched with SFA. This effect was potentiated when these animals had been previously subjected to protein restriction diet during critical periods of development (Figure 5F). However, no changes were noted in the transcriptional levels of GRP75, showing that the transcriptional level of VDAC and GRP75 are affected differently by the nutritional transition evaluated in the present study. The study of of Le, et al. [79] showed greater VDAC1 expression in mice liver fed with HFD by 16 weeks. The authors suggest that VDAC1 inhibition may be an underlying mechanism of drugs, such as sennoside A, a commonly used clinical laxative stimulant, for protecting mitochondria in HFD-induced hepatic steatosis in mice. VADC may be a promising target for treating fatty liver disease [79].

Mfn2 [80] and CypD [81] have also been cited as involved in the interactions between ER and mitochondria. Loss of these proteins affects the interactions and induces ER stress. However, the interactions can increase acute responses to ER stress, leading to increased mitochondrial respiration and Ca^2+^ accumulation with swelling and dysfunction [82]. One of our hypotheses tested after the mitochondrial function essays was in what way the nutrition transition from perinatal protein restriction to a post-weaning SFA-enriched diet would affect mitochondrial respiration and swelling, at least in part, by altering the transcriptional levels of these proteins. The data obtained in this paper, however, indicated that the harmful effects observed in mitochondrial bioenergetic functions seems not to be related to dysfunction at transcriptional levels of Mfn2 and CypD. In order to evaluate detail in this process, additional and specific experiment will be needed.

## 5. Conclusions

This study showed that SFA-enriched HFD induces mitochondrial dysfunction in the liver of young rats, and that this effect was potentiated when the animals were submitted to a low protein diet during critical periods of development. Our results suggest that this mitochondrial dysfunction is associated with oxidative stress, greater expression of VDAC that potentiates the opening of the mitochondrial permeability transition pore, and lower mitochondrial biogenesis.

## Figures and Tables

**Table 1:** Sequences of primers used for the RT-PCR analysis.

Gene Sequence	F/R	5′-3′	Tm (°C)	Amplicon Size	Ref NCBI
Tbp	F	TGGTGTGCACAGGAGCCAAG	62	139pb	NM_001004198
	R	TTCACATCACAGCTCCCCAC			
Mfn2	F	TTGGATGGACTATGCTAGTG	60	230pb	NM_130894
	R	TCCTCCGACCACGAGAATG			
Hspa9 (Grp75)	F	TGATGCCAATGGGATTGTGC	60	175pb	NM_001100658
	R	CTGCTTCAACACGTTCCTTC			
Ppif (CypD)	F	GGCTACAAAGGCTCCACCTTC	62	112pb	NM_172243
	R	GAAAGCGGCTTCCGTAGATG			
Vdac1	F	AACAGTAACACTCGCTTTGG	60	167pb	NM_031353
	R	TTGACGTTCTTGCCATCCAG			
Tfam	F	GCTTGGAAAACCAAAAAGAC	60	201pb	NM_031326
	R	CCCAAGACTTCATTTCATT			
Pgc-1α	F	TCCTCTGACCCCAGAGTCAC	60	143pb	NM_031347
	R	CTTGGTTGGCTTTATGAGGAGG			

mRNA expression levels were quantified from liver-derived cDNAs for the following genes: TATA box binding protein (Tbp; housekeeping), mitofusin 2 (Mfn2); Heat shock protein 9 (Hspa9); Peptidylprolyl isomerase F (Ppif); voltage-dependent anion channel 1 (Vdac); Transcription factor A, mitochondrial (Tfam); Peroxisome proliferator-activated receptor gamma, coactivator 1 alpha (Pgc-1α).
